# MiR-146a polymorphism correlates with lung cancer risk in Chinese nonsmoking females

**DOI:** 10.18632/oncotarget.13722

**Published:** 2016-11-30

**Authors:** Zhihua Yin, Zhigang Cui, Yangwu Ren, Lingzi Xia, Hang Li, Baosen Zhou

**Affiliations:** ^1^ Department of Epidemiology, School of Public Health, China Medical University, Shenyang, PR China; ^2^ Key Laboratory of Cancer Etiology and Intervention, University of Liaoning Province, Shenyang, PR China; ^3^ School of Nursing, China Medical University, Shenyang, China

**Keywords:** lung cancer, microRNA, single nucleotide polymorphism, genetic susceptibility, gene expression

## Abstract

This study provides evidence that the common rs2910164 polymorphism in miR-146a strongly correlates with lung cancer risk in nonsmoking females in northeast China. The genotypes of miR-146a rs2910164 were determined in 1131 patients with lung cancer and 1003 healthy control subjects. Tissue samples were used to evaluate the association between miRNA expression and lung cancer risk as well as the correlation between rs2910164 genotypes and miR-146a expression. The secondary structures of the wild-type and variant miR-146a sequences were predicted, and luciferase-based target assays were used to test whether miR-146a bound to tumor necrosis factor receptor associated factor 6 (TRAF6) mRNA. Individuals carrying heterozygous CG genotype of miR-146a rs2910164 had less risk of lung cancer than those carrying homozygous wild CC genotype (OR = 0.76, 95% CI = 0.60-0.98, *P* = 0.032). We found no significant association between miR-146a expression and lung cancer risk. MiR-146a expression differed in those carrying the CC genotype as compared with the CG or the GG genotype (*P* = 0.032 and 0.001), and the secondary structure of the C allele differed slightly from the G allele. Significantly lower levels of luciferase activity were observed when the TRAF6 3′UTR was cotransfected with miR-146a-3p carrying the rs2910164 C allele (*P* = 0.001). Thus, miR-146a rs2910164 polymorphism may influence susceptibility to lung cancer in Chinese nonsmoking females through targeting TRAF6.

## INTRODUCTION

Lung cancer is the most common malignant tumor, and causes approximately 1.38 million deaths worldwide each year [1]. In China, the incidence and mortality of lung cancer has increased significantly in the past two decades and is the most common malignant tumor in urban populations [2].

Although cigarette smoking is considered the predominant risk factor for lung cancer, nonsmokers account for an estimated 15% and 53% of male and female lung cancer patients, respectively [3]. In addition to tobacco, other risk factors contribute to the etiology of lung cancer. Therefore, nonsmokers are the ideal population in which to study the epidemiologic characteristics and risk factors of lung cancer other than cigarette smoking. Lung cancer rates in Chinese women (20.4 cases per 100,000 women) are higher than rates among women in some European countries, despite a lower prevalence of smoking [4]. The incidence of lung cancer among women is approaching the peak incidence in men. A report by Wakelee et al [5] suggests that female nonsmokers are more likely than male nonsmokers to develop lung cancer. Thus, study of the factors for lung cancer in females is urgently needed. Nonsmoking females are the subjects in this study of the etiology of lung cancer.

Molecular epidemiologic studies have shown that hundreds of genes are involved in lung carcinogenesis, including previously known genes such as p53, Rb, and Ras and newly developed markers [6-7]. Although the previously known genes might yield further understanding of lung cancer development, newly developed markers such as noncoding small RNAs might lead to new understanding of the molecular mechanisms of lung cancer [8]. MicroRNAs (miRNAs) are a class of 21- to 24-nucleotide noncoding RNA gene products that are believed to regulate gene expression by binding to *cis*-acting regulatory elements of the 3′ untranslated region (UTR) of target genes, leading to inhibition of the translation of the latter, thus disrupting the expression of the protein [9]. MiRNAs are implicated in the regulation of such crucial biological processes as development, differentiation, apoptosis, and proliferation, and various diseases, including cancer [10]. More than 50% of miRNA genes are in cancer-associated genomic regions or in fragile sites, suggesting that miRNAs are important factors in the pathogenesis of human cancers [11].

Studies show that single nucleotide polymorphisms (SNPs) in miRNA-containing genomic regions might significantly alter the production or processing of miRNA [12-13]. The SNPs in an miRNA sequence might influence expression, secondary structure and/or target genes of miRNA and consequently modify cancer risk.

In our previous case-control studies, the correlation of the SNPs in pre-miRNAs with risk of lung cancer in nonsmoking females in northeast China was shown for SNP rs2910164 in miR-146a. However, no function results were shown [14-16]. In the present study, more cases and control subjects have been added to confirm the association between miR-146a polymorphism and lung cancer risk and effects on miRNA expression, secondary structure, and target gene regulation.

## RESULTS

The present study includes 1131 cases and 1003 control subjects, among which are 575 cases and 608 control subjects whose results have been reported [14]. Therefore, the case-control study results in the present study are defined as first-stage (published results) and second-stage (new added results) (Table 1). All individuals were nonsmoking females. The mean ages were 56.94 ± 11.72 years and 56.27 ± 12.56 years in the case group and the control group, respectively. There were no differences in ages between cases and control subjects (*P* = 0.200). Among lung cancer cases, 761 patients had adenocarcinoma, 197 had squamous cell carcinoma, and 166 had other types of cancer. The genotype distributions of miR-146a rs2910164 SNP in the cases and control subjects are shown in Table 2. The observed genotype frequencies agree with those expected under the Hardy-Weinberg equilibrium in the control subjects (*P* = 0.521).

**Table 1:** Allele and genotype frequencies of miR-146a polymorphism among cases and control subjects in nonsmoking female population

SNP	First stage				Second stage			
	Cases (%)	Control subjects (%)	OR (95%C1)	*P* value	Cases (%)	Control subjects (%)	OR (95%CI)	*P* value
CC (ref)	198 (34.4)	168 (27.6)	1.00 (ref)		179 (32.2)	122 (30.9)	1.00 (ref)	
CG	280 (48.7)	313 (51.5)	0.76 (0.59-0.99)	0.039	270 (48.6)	195 (49.4)	0.94(0.70-1.27)	0.700
GG	97(16.9)	127 (20.9)	0.65 (0.46-0.91)	0.011	107(19.2)	78(19.7)	0.94 (0.65-1.36)	0.723
Dominant model			0.73 (0.57-0.93)	0.012			0.94 (0.71-1.24)	0.669
CG+GG vs								
CC								
Recessive			0.77 (0.57-1.03)	0.078			0.97 (0.70-1.34)	0.847
model GG								
vs CC+CG								
C allele (ref)	676 (58.8)	649 (53.4)	1.00 (ref)	—	628 (56.5)	439 (55.6)	1.00 (ref)	—
G allele	474 (41.2)	567 (46.6)	0.80 (0.68-0.94)	0.008	484 (43.5)	351 (44.4)	0.96 (0.80-1.16)	0.695

**Table 2:** The association of miR-146a polymorphism and lung cancer risk

SNP	Cases(%)	Control subjects (%)	OR (95%CI)	*P* value	OR (95%CI)*	*P* value
CC(ref)	377 (33.3)	290 (28.9)	1.00 (ref)		1.00 (ref)	
CG	550 (48.6)	508 (50.6)	0.83 (0.69-1.01)	0.066	0.83 (0.69-1.01)	0.068
GG	204(18.0)	205 (20.4)	0.77 (0.60-0.98)	0.034	0.76 (0.60-0.98)	0.032
Dominant model CG+GG vs CC			0.81 (0.68-0.98)	0.028	0.81 (0.68-0.98)	0.028
Recessive model GG vs CC+CG			0.86(0.69-1.06)	0.160	0.85 (0.69-1.06)	0.148
C allele (ref)	1304 (57.6)	1088 (54.2)	1.00 (ref)	—		
G allele	958 (42.4)	918 (45.8)	0.87 (0.77-0.98)	0.025		

Abbreviation: SNP, single nucleotide polymorphism; OR, odds ratio; CI, confidence interval. *ORs were calculated by unconditional logistic regression and adjusted for age.

Table 2 shows the association between miR-146a rs2910164 SNP and lung cancer risk. Individuals carrying homozygous GG genotype have decreased risk of lung cancer compared with the homozygous wild CC genotype (adjusted OR is 0.76, 95% CI = 0.60-0.98, *P* value is 0.032). In the dominant model, the combination of heterozygote CG and variant homozygote GG for rs2910164 was associated with a significantly reduced risk of lung cancer compared with its wild-type homozygote CC (adjusted OR = 0.81, 95% CI = 0.68-0.98, *P* = 0.028). Allele comparison showed that the G allele was associated with a lower risk of lung cancer, with a significant OR of 0.87 (95% CI= 0.77-0.98, *P* = 0.025). Further analyses were performed to stratify cancer by pathological type, and similar significant results were found in the lung adenocarcinoma group but not in the squamous cell lung cancer group (Table 3).

**Table 3:** The association of miR-146a polymorphism and lung cancer risk by cancer type

SNP	OR (95%CI)	*P* value	OR (95%CI)*	*P* value
**Adenocarcinoma**				
CC(ref)	1.00 (ref)		1.00 (ref)	
CG	0.82 (0.66-1.01)	0.061	0.82 (0.66-1.01)	0.063
GG	0.71 (0.54-0.93)	0.014	0.71 (0.54-0.93)	0.013
Dominant model	0.78 (0.64-0.96)	0.018	0.78 (0.64-0.96)	0.019
CG+GG vs CC				
Recessive model GG	0.80 (0.63-1.02)	0.075	0.80 (0.63-1.02)	0.070
vs CC+CG				
C allele(ref)	1.00 (ref)			
G allele	0.84 (0.73-0.96)	0.011		
**Sq**				
CC(ref)	1.00 (ref)		1.00 (ref)	
CG	0.83 (0.59-1.18)	0.295	0.84 (0.59-1.19)	0.328
GG	0.88 (0.57-1.36)	0.578	0.89 (0.57-1.37)	0.582
Dominant model	0.85 (0.61-1.17)	0.315	0.85 (0.61-1.19)	0.342
CG+GG vs CC				
Recessive model GG	0.99 (0.68-1.45)	0.966	0.99 (0.67-1.44)	0.939
vs CC+CG				
C allele(ref)	1.00 (ref)	—		
G allele	0.93 (0.75-1.15)	0.499		

Abbreviation: SNP, single nucleotide polymorphism; OR, odds ratio; CI, confidence interval. *ORs were calculated by unconditional logistic regression and adjusted for age.

**Figure 1:**
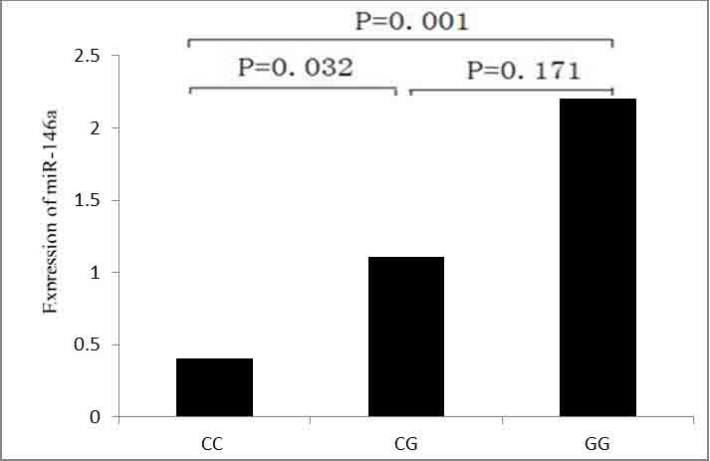
Sequence variations in the miR-146a can influence its expression The relative expression (2-^△△^Ct) of miR-146a to U6 was significantly different between the CC genotype and the CG or GG genotype.

The characteristics of 60 study subjects for quantitative real-time RT-PCR were as follows: age, 55.9 ± 1.41 years; adenocarcinoma, 24 subjects; squamous cell cancer, 22 subjects; and other types of cancer 14 subjects. The △△Ct values of the control subjects and cases were 0.00 ± 2.68 and −0.11 ± 2.22, suggesting no significant association between miR-146a expression and lung cancer risk (*t* = 0.245, *P* = 0.807). To further characterize the functional relevance of the miR-146a rs2910164 SNP, we conducted correlation analysis between rs2910164 genotypes and the expression of miR-146a. Among these specimens, 13 were of the rs2910164 CC genotype, 29 were of the CG genotype, and 18 were of the GG genotype. The △△Ct values were 1.29 ± 1.25 for the rs2910164 CC genotype; −0.15 ± 2.09 for the CG genotype; and −1.14 ± 2.52 for the GG genotype . As shown in Figure 1, the relative expression of miR-146a to U6 was significantly different between the CC genotype and the CG and GG genotypes (*P* values were 0.032 and 0.001, respectively). Therefore, the increased relative expression of miR-146a (lower Ct ratio corresponds to a higher expression) in rs2910164 CG or GG carriers was associated with potentially enhanced processing from pre-miR-146a to its mature form (Figure 1).

**Figure 2:**
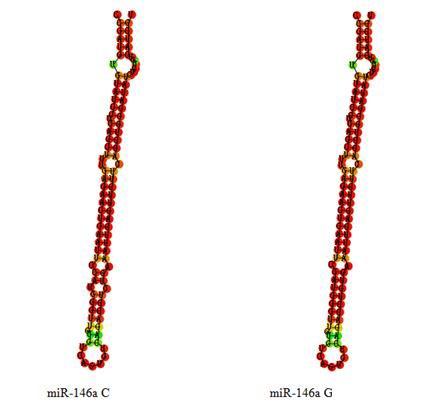
Sequence variations in the miR-146a can translate into structural alterations The RNA secondary structure was predicted by RNAHYbrid. Only the most stable secondary structures with the lowest free energy are depicted.

We tested whether such variants could affect miR-146a secondary structure and thereby block processing into the functional mature miR-146a. We observed a slight secondary structure change in the C allele compared with the G allele from the predicted secondary structures (Figure 2). The G allele destroyed a base-pairing, which disrupted the integrity of the stem and changed the secondary structure of miR-146a. The optimal free energies were −38.80 Kcal/mol for the C allele and −41.80 Kcal/mol for the G allele, suggesting a less stable secondary structure for the C allele than the G allele. Similarly, genetic variants might affect miR-146a secondary structure and thereby alter its processing.

We identified the target genes of miR-146a by combining the search results in Targetscan, the probability of interaction by target accessibility (PITA), and the European Molecular Biology Laboratory (EMBL). We studied tumor necrosis factor receptor associated factor 6 (TRAF6).

**Figure 3:**
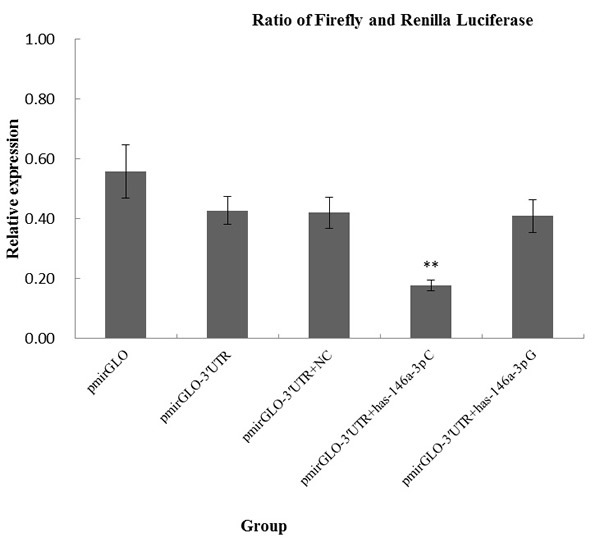
*In vitro* target binding assays for rs2910164 in A549 cell lines Each transfection was performed with pRL-SV40 plasmids as normalizing controls. Data presented are the mean fold increase ± SD from 3 independent transfection experiments, and each was done in triplicate.

To evaluate the influence of the rs2910164 variant on the binding of miR-146a-3p to TRAF6 mRNA, we generated TRAF6 3′UTR luciferase reporter plasmids that were cotransfected with chemically synthesized mature miR-146a-3p miRNAs (C or G allele) in A549 cell lines. As shown in Figure 3, we observed significantly lower levels of luciferase expression when we cotransfected TRAF6 3′UTR luciferase reporter plasmids with chemically synthesized mature miR-146a-3p miRNA carrying the rs2910164 C allele (0.177 ± 0.018 for C versus 0.409 ± 0.054 for G, *P* = 0.001). The results suggested that miR-146a-3p wild-type might be able to specifically combined to TRAF6 3′UTR and significantly inhibit the translation of TRAF6 gene.

## DISCUSSION

This study is the first to provide evidence that common SNP rs2910164 in miR-146a might be an important factor in lung cancer risk through disrupting miR-146a expression and secondary structure as well as directly inhibiting the target gene TRAF6. Our findings suggest that the rs2910164 CG/GG genotypes in miR-146a were associated with deceased risk of lung cancer, particularly among adenocarcinoma patients. The association between miR-146a rs2910164 polymorphism and risk of several malignancies were shown to be inconsistent by some results. The heterozygous genotype (CG) increased the risk of papillary thyroid cancer [17]. Shen et al [18] found that the C allele was associated with the increased risk of early onset familial breast and ovarian cancers. The C allele was suggested to be a risk allele for gastric cancer in a Japanese study [19]. The GG genotype was reported to correlate with increased risk for hepatocellular carcinomas, esophageal squamous cell carcinoma, cervical cancer and gastric cancer [20-23]. Xu et al [24] found that the CC genotype was associated with a decreased prostate cancer risk. Jeon et al [25] found that individuals carrying the CG or GG genotype were less likely to develop cancer compared with the CC genotype in a Korean population [25], which is similar to the present study results.

Because the minor allele frequency of rs2910164 is significantly different among different ethnic populations (from 0.26 to 0.80), its relation to lung cancer in diverse ethnic populations should be studied. Our study is one of two published studies on the relation between miR-146a rs2910164 polymorphism and lung cancer risk in Chinese [14, 26]. We have undertaken a series of studies in Chinese nonsmoking females that could reduce the confounding bias caused by population stratification.

The control group in our study comprised only healthy subjects who were free of other diseases that might be associated with miRNA SNP. We could therefore exclude the effects of other diseases on the genotype distributions in the control subjects. The genotype distribution for rs2910164 was accordant with Hardy-Weinberg equilibrium (HWE), further supporting the randomness of our control subjects. The present study included a relatively large number of cases and control subjects. These conditions increase the reliability of our findings.

MiRNA expression patterns have been suggested to correlate with the biological and clinical behavior of human solid tumors [8, 27-28]. For example, the miR-146a expression was significantly decreased in non-small cell lung cancer (NSCLC) patients compared with that of nonmalignant lung tissues [29]. Serum levels of miR-146a were significantly lower than those in control subjects [30], whereas serum miR-146a was overexpressed in NSCLC patients compared with healthy control subjects [31]. We found no significant association between miR-146a expression and lung cancer risk. The present study also showed that SNP rs2910164 could increase miR-146a expression levels [29], and suggested that patients carrying a variant homozygote of miR-146a rs2910164 SNP had a lower risk of lung cancer, possibly through a mechanism of elevated expression of miR-146a and weakened target binding of miR-146a-3p.

 The change of the miRNA secondary structure caused by SNP or mutation has been hypothesized to influence the maturation process of miRNA We found that the G to C SNP of miR-146a rs2910164 caused a structure disturbance in the stem region of the miR-146a and leads to the increased production of mature miR-146a. Similar results have also been found for other miRNA genes. The SNPs of miR-125a and miR-30 could introduce base-pairing mismatches, alter free energy values, and create large bulges in the predicted secondary structure, which are detrimental to the production of miRNAs [12].

The gene regulation results in this study found that lung cancer patients with the miR-146a rs2910164 CC genotype expressed significantly lower levels of TRAF6 and suggested that miR-146a-3p wild-type might be able to specifically combined with TRAF6 3′UTR and significantly inhibit the translation of TRAF6 genes. Published results have identified the genetic modifier for the development of cancer using the same research approach, including association between noncoding region SNPs and cancer risk, expression study, bioinformatics analyses, and dual luciferase reporter gene assays [32-33].

This case-control study has some limitations. First, the cases and control subjects were recruited from hospitals, which might result in hospital-based Berkson's bias. To decrease the possibility of Berkson's bias, cases and control subjects were chosen from several different hospitals. Furthermore, the control subjects were healthy individuals recruited from medical examination centers in the hospitals. Second, limited information on the environmental exposure of study subjects restricted gene-environment interaction analysis. Third, the involvement of additional SNPs and/or regulatory markers that are yet to be discovered might be a confounding factor. A study comprising additional SNPs and/or related molecular markers with a larger population would provide further clarification.

Our study provides evidence that polymorphism in miR-146a rs2910164 C>G might alter individual susceptibility to lung cancer through inhibiting miR-146a expression and secondary structure, as well as directly influencing the target gene TRAF6. Future larger studies with other ethnic populations and male lung cancer patients are required to confirm the current findings.

## CONCLUSIONS

The miR-146a rs2910164 C>G polymorphism might contribute to genetic susceptibility to lung cancer in Chinese nonsmoking females through affecting miR-146a expression and secondary structure, as well as directly influencing the target gene TRAF6.

## MATERIALS AND METHODS

### Study subjects

This study is an ongoing lung cancer study in nonsmoking females in Shenyang City, located in northeast China. The study subjects consisted of 1131 lung cancer patients and 1003 healthy control subjects (between July 2010 and December 2014). The inclusion and exclusion criteria of lung cancer cases and the control subjects were the same as reported previously [14]. The human investigations were approved by the Institutional Review Board of China Medical University, and informed consent was obtained from each participant. Each participant donated 10 mL of venous blood for SNP detection at the time she was admitted to the hospital. An individual with a total of 100 cigarettes in her lifetime was defined as a smoker, otherwise she was considered as a nonsmoker.

### SNP genotyping

Genomic DNA samples were extracted by the Phenol-chloroform method. The samples were read and analyzed by the ABI 7500 Fast Sequence Detection System that used SDS 4.2.3 software (Applied Biosystems, Lifetechnologies, USA). The TaqMan allelic discrimination method was used to genotype miR-146a SNP rs2910164 per the protocol provided with a commercially available primer probe set (assay ID C_15946974_10). Quality control was conducted by randomly selecting 10% of samples for repeat genotyping, and the results were concordant for all the duplicate sets.

### Quantitative RT-PCR

To determine the expression levels of miRNA, tissues were obtained from patients who had undergone resection for lung cancer. Sixty lung cancer tissues and the accompanying control tissues were used to evaluate the association between miRNA expression and lung cancer risk. Control tissues were surgically resected normal tissues adjacent to the tumors of lung cancer patients. Three groups of tissues for three types of rs2910164 genotypes were compared to evaluate the correlation between rs2910164 genotypes and the expression of miR-146a. Total RNA was extracted by the Trizol method. TaqMan MicroRNA Reverse Transcription Kit and TaqMan MicroRNA Assays (Applied Biosystem, Lifetechnologies, USA) were utilized to reverse transcribe RNA to cDNA and quantify mature miRNA levels. The PCR reaction was repeated three times for each sample, and U6 was used as an internal reference. The comparative CT method and △△CT and 2^-△△CT^ were used to analyze the miRNA expression level. Real-time PCR was performed on ABI 7500 Fast Sequence Detection System (Applied Biosystems, Lifetechnologies, USA).

### Primary precursor of miRNA secondary structure predictions and optimal free energy calculations

Secondary structures for the wild-type and variant primary precursor of miR-146a sequences were predicted by use of RNAhybrid (http://bibiserv.techfak.uni-bielefeld.de/rnahybrid/submission.html). The minimum optimal free energies for wild-type and variant miR-146a were calculated by use of RNAhybrid. The predictions and calculations were made per the instructions, and the default setting of the program was used.

### Target *in vitro* assay

Luciferase-based target *in vitro* assay was applied to test whether miR-146a could bind to the 3′UTR of TRAF6 mRNA. The 3′UTR segments of TRAF6 mRNA predicted to interact with miR-146a were amplified by PCR from human genomic DNA. A 454-bp fragment of the 3′UTR segments of TRAF6 was amplified by use of the following primers: 5′-CAGGGCTAGCTACTTTCTTGGGCTTTTGCT and 5′-CGCAGTCGACAGATGCTACTTCGTAACCTCA. The PCR fragment was inserted into the pUM-T simple vector (BioTeke). The insertion of the fragment was confirmed by sequence analysis. Mimics of miR-146a-3p and negative control were generated as follows: CCUCUGAAAUUCAGUUCUUCAG, CCUGUGAAAUUCAGUUCUUCAG, and UUCUCCGAACGUGUCACGUTT. Then, pmirGLO-TRAF6 was transfected into the A549 lung cancer cell line with or without pcDNA-miR-146a using Liposomal Transfection reagents per the instructions. The cells were inoculated in 96-well plates and divided in five groups: pmirGLO, pmirGLO-3′UTR, pmirGLO-3′UTR+NC, pmirGLO-3′UTR+miR-146a-3p-C allele, pmirGLO-3′UTR+miR-146a-3p-G allele. Luciferase activities were measured by application of luciferase assays (Promega, Madison, WI) 24 hours after transfection. The levels of interaction between TRAF6 and miR-146a were determined by the difference of luciferase activities between the cells. Each experiment was triplicated and the mean of the triplicates was used. Luciferase assays were performed by use of the Dual-Luciferase reporter assay systems (Promega) and Lumat LB9507 (Berthold, Germany).

### Statistical analysis

In the case-control study, Pearson's χ^2^ test and the Student *t* test were performed to examine differences in categorical and continuous variables, respectively. HWE of the SNP was analyzed by the goodness-of-fit χ^2^ test. The OR and their 95% CI were calculated by unconditional logistic regression analyses to assess the association between miR-146a SNP and lung cancer risk. The Student *t* test was used to compare mean expression levels for differences between the wild-type and variant allele of miR-146a in the miRNA expression assay, and compare the mean luciferase activities between cells. A statistical significance was defined as *P* < 0.05, and all the statistical tests were two-sided. All analyses were performed by SPSS software (Version 20.0, IBM SPSS, Inc. Chicago, IL, USA).
